# Mapping the variability of *in vivo* rat uterine electrical properties throughout the oestrous cycle

**DOI:** 10.1113/EP092704

**Published:** 2025-08-21

**Authors:** Mathias W. Roesler, Amy S. Garrett, Leo K. Cheng, Alys R. Clark

**Affiliations:** ^1^ Auckland Bioengineering Institute The University of Auckland Auckland New Zealand

**Keywords:** electrical activity, electrophysiology, oestrous cycle, uterus

## Abstract

After reaching sexual maturity, uterine function is driven by cyclical variations in hormone levels. The electrical and mechanical activity in the uterus varies during the menstrual cycle, contributing to essential functions such as sperm transport and shedding the menstrual lining. However, there is a lack of quantification of the variation in uterine function occurring over the course of the cycle. Female Wistar rats were used to quantify the changes in uterine electrical activity *in vivo* with respect to the oestrous cycle. Under anaesthesia, the uterus was exteriorised, and electrodes were placed on the dorsal and ventral sides of the organ to record spontaneous activity from the serosa. Electrical events were separated into slow and fast components based on frequency. The duration and interval between events were measured and propagation directions and velocities were mapped along the uterus using high spatial resolution electrode arrays. All stages of the oestrous cycle showed ovarian–cervical propagation, but cervical–ovarian propagation was also present in pro‐oestrus and metoestrus. Ovarian‐cervical propagation was dominant in oestrus and metoestrus. The interval between events showed significant differences with 40.2 ± 5.6 s (1.51 ± 0.25 cpm) and 60.5 ± 2.6 s (1.02 ± 0.05 cpm) during the dioestrus and metoestrus phases, respectively (*P* < 0.001). The slow and fast component durations were similar across the oestrous cycle (19.9 ± 2.2 s and 10.2 ± 3.0 s, respectively). This emphasizes the role of the oestrous cycle in guiding uterine function through modulation of the electrical activity and shows potential for estimating the oestrous cycle phases based on electrical characteristics.

## Abstract (highlights)

- **What is the central question of this study?**
  How do the spatial patterns of electrical activity in the uterus vary over the oestrous cycle in rats?
- **What is the main finding and its importance?**
  High‐resolution mapping of the electrical activity in the rat uterus showed variation in the propagation patterns and directions across the stage of the oestrous cycle. High‐frequency electrical events are typically associated with contractions, but low‐frequency activities (slow waves) are not always accompanied by these high‐frequency events, suggesting research and clinical studies should focus on both components of electrical activity as indicators of uterine function.

## 1 INTRODUCTION

The uterus is a smooth muscle organ displaying varying patterns of electrical and mechanical activity at different phases of its hormonal cycle (van Gestel et al., 2003). Although Bozler measured spontaneous electrical activity in uterine tissue for the first time in 1938 (Bozler, 1938), there remain gaps in our understanding of uterine function (Garrett et al., 2022). The function and direction of uterine contractions vary across the menstrual cycle. For example, contractions moving from the ovaries to the cervix may help expel the menstrual lining in menstruating animals such as humans, but contractions in the opposite direction could benefit sperm mobility towards the ovaries during fertilisation (Wray & Prendergast, 2019). Uterine smooth muscle cells undergo spontaneous contractions via the generation of action potentials. This occurs when the membrane potential (–80 to –40 mV) is depolarised enough to trigger the opening of voltage‐dependent ion channels in the cell membrane. Of note, the voltage‐gated L‐type calcium channels, which activate at approximately –40 mV in humans (–30 mV in rats), trigger an influx of calcium which activates the contractile apparatus in the cell. The calcium influx comes from the extracellular space via transmembrane channels and from internal calcium stores (sarcoplasmic reticulum) (Wray & Prendergast, 2019). Electrical activity in the uterus is therefore critical for generating the necessary contractions for reproduction and uterine health. Moreover, dysfunctional activity has been linked to chronic health issues such as endometriosis, dysmenorrhoea and infertility (Kunz & Leyendecker, 2002).

Steroid hormones, notably oestrogen and progesterone, drive variations in uterine function (Wray & Noble, 2008). Concentrations of these hormones vary throughout the oestrous cycle and trigger changes in anatomy, mechanical and electrical activity (Westwood, 2008; Wray & Noble, 2008). However, measuring the changes in uterine electrical activity directly in humans can be difficult due to challenges in performing invasive recordings directly from the uterus and the low signal‐to‐noise ratios of signals recorded from the surface of the body. Previously, Kuijsters et al. (2020) recorded spontaneous electrical activity *ex vivo* in human uteri after a hysterectomy for hypermenorrhoea or dysmenorrhoea. They were able to measure the propagation of electrical events over the surface of the uterus. Most of the events propagated erratically; however, it is difficult to know if this is due to the menstrual cycle, the underlying pathological condition or the experimental conditions. Non‐invasive methods have been recently applied using electrodes on the surface of the body; however, the signals are attenuated, often making interpretation challenging (Wang et al., 2024). Nevertheless, the measured electrical signals showed that the dominant propagation direction changed during the cycle. During menses, the primary direction was fundo‐cervical, while during the peri‐ovulatory phase, it was cervico‐fundal. The peri‐ovulatory phase exhibited the most activity compared to the other three phases of the menstrual cycle. However, transvaginal ultrasound was used as validation, which has been shown to alter uterine activity (Anderson et al., 2024). Laboratory animals, such as rats, can be used to measure *in vivo* signals directly on the surface of the uterus allowing for high‐resolution and high‐quality recordings, and direct correlation between electrical signals and contraction patterns (Garrett, Roesler et al., 2024).

In rats, the oestrous cycle lasts between 4 and 5 days and consists of four stages: pro‐oestrus, oestrus, metoestrus and dioestrus (Ajayi & Akhigbe, 2020). Previous attempts to quantify the uterine electrophysiology properties outside of pregnancy are sparse, and the results are conflicting (Crane & Martin, 1991; Ishikawa & Fuchs, 1978; Talo & Kärki, 1976). The stages of the oestrous cycle were classified differently in these studies: in Talo & Kärki (1976), the pro‐oestrus and dioestrus phases were both subdivided into two further phases, in Ishikawa & Fuchs (1978), the metoestrus phase was not included, whereas in Crane & Martin (1991), the four principal stages (mentioned above) were reported. Nevertheless, these studies report similar metrics, namely, propagation direction, duration and frequency of electrical events. Recordings were performed over the entire oestrous cycle in conscious rats after surgically implanting three to six electrodes at different locations in the uterus (Crane & Martin, 1991; Ishikawa & Fuchs, 1978; Talo & Kärki, 1976). In all three studies, the majority of events propagated in the cervical–ovarian direction, and the oestrus phase of the cycle displayed the highest frequency of events. Although the comparison between these studies is limited by the reporting of oestrus, they clearly show that it affects the electrical and mechanical activity of the organ.

There has been recent renewed interest in non‐pregnant uterine electrical activity with studies performed on sows and rats *in vivo*. Domino et al. (2018, 2016) and Rutkevich et al. (2025) used a limited number of bipolar electrodes to record uterine activity, whereas Garrett, Roesler et al. (2024) used high‐resolution electrodes to cover the uterine surface. Electrode array mapping has previously been applied in the gastrointestinal system (Athavale et al., 2024) and pioneered *ex vivo* in the pregnant uterus by the Lammers group (Lammers et al., 2015; Lammers & Hamid, 1998; Lutton et al., 2018), where they characterised contraction types and possible locations of pacemaking activity. Garrett, Roesler et al. (2024) showed that uterine electrical activity in the non‐pregnant rat uterus comprises two components occurring concomitantly: high‐frequency (fast wave made of a burst of activity) and low‐frequency (slow wave). To build on this work, the variation of electrical properties of electrical activity over the course of the oestrous cycle were considered. Both high and low frequency components were identified in the electrical activity. Using electrode arrays with higher spatial resolution made it possible to quantify the propagation properties and patterns in all four stages of the oestrous cycle. Additionally, the relationship between the two components was explored by evaluating the number of events exhibiting both high and low frequency components; finally, the duration and interval between electrical events were measured.

## 2 METHODS

### 2.1 Ethical approval

Ethical approval was granted by the University of Auckland Animal Ethics Committee (AEC23005, AEC25868), and all exp erimental protocols were carried out in accordance with institutional animal ethics guidelines and comply with the animal ethical principles under which the journal operates. A total of 19 virgin non‐pregnant female Wistar rats at different stages of the oestrous cycle (*n* = 5 in pro‐oestrus, metoestrus and dioestrus, *n* = 4 in oestrus) were used (12.9 ± 0.6 weeks old, 266.6 ± 18.4 g). Animals were purchased from the animal facility (Vernon Jansen Unit) at the University of Auckland, where they were housed in a 12/12 light—dark cycle at room temperature (21–22°C) and fed standard rat chow and tap water ad libitum. For each experiment, the rat was anaesthetised with isoflurane mixed with 100% medical oxygen (4–5% via an induction box) and anaesthesia was maintained (1.5–2.5% via a nose cone) throughout the experiment. Depth of anaesthesia was determined via pedal withdrawal in conjunction with observation of heart rate, respiratory rate and depth, colour of the skin, ears and tail, and response to surgical stimulus overall. Heart rate and body temperature were monitored and maintained within physiological limits. Upon completion of the experiment, the rat was euthanised via cervical dislocation while still under anaesthesia.

### 2.2 Experimental set‐up

**FIGURE 1:**
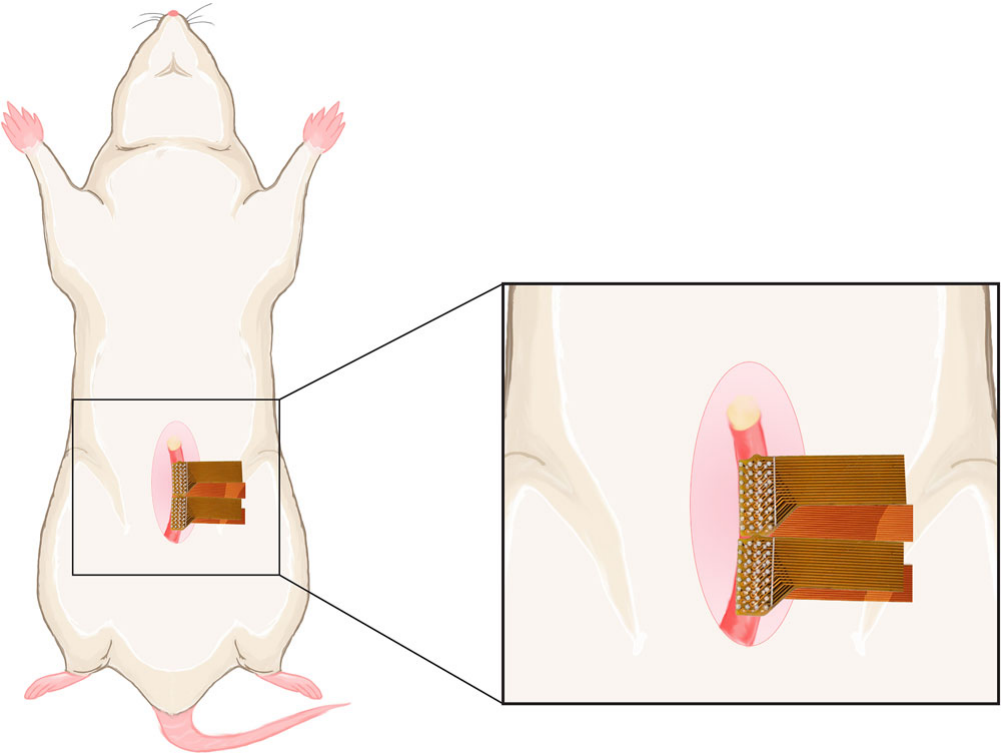
Experimental set‐up for measuring the electrical activity of the uterus. The organ was exposed while the animal was under anaesthesia. Flexible electrode array pairs were taped together and placed on the ventral and dorsal sides of the uterine serosa. Spontaneous electrical activity was recorded with an ActiveTwo acquisition system (BioSemi, Amsterdam, the Netherlands).

The experimental set‐up has been previously described in detail (Garrett, Roesler et al., 2024). In brief, upon induction of anaesthesia, a midline laparotomy was performed to expose the uterus. Flexible printed circuit electrode arrays were carefully placed on the ventral and dorsal sides of the uterine serosa (Athavale et al., 2024). Two electrode arrays, each consisting of 32 electrodes arranged in an 8‐by‐4 grid with a 0.95 mm inter‐electrode spacing, were taped together and covered 16 mm of the length of the uterine horn. A schematic diagram of the experimental set‐up is shown in Figure 1. Once in place, the uterus was covered with plastic wrap to help maintain moisture and temperature. A heat lamp was used to increase the body temperature if it fell below 36.5°C. The electrodes recorded spontaneous activity at a sampling rate of 512 Hz using an ActiveTwo acquisition system (BioSemi, Amsterdam, the Netherlands) but were subsequently down‐sampled to 30 Hz for computational efficiency. Reference electrodes were positioned on each thigh. Each recording lasted approximately 10 min.

### 2.3 Oestrous staging

At the conclusion of each study, the stage of the oestrous cycle was determined via vaginal cytology (McLean et al., 2012). A pipette with distilled water was used to perform a vaginal lavage and recuperate cells from the vagina. The fluid in the pipette was placed on glass microscope slides and air‐dried. Once dry, the slides were placed in a solution of 0.1% crystal violet for 1 min to stain the cells. Excess staining solution was removed by placing the microscope slides in distilled water for 1 min. The slides were carefully dried with a tissue and placed under a microscope with ×20 magnification to view the cells and determine the stage of the oestrous cycle.

### 2.4 Data processing

**FIGURE 2:**
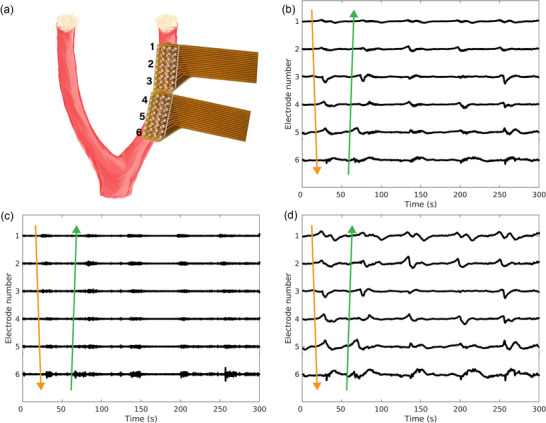
Representative traces of normalised electrical signals measured along the uterine horn from a single rat. The traces were extracted from six electrodes along one column of the arrays and normalised. (a) Diagram indicating the placement of the electrode arrays on the uterine horn and the selected channels displayed in the other panels. (b) The traces of the signals after the baseline drift had been removed. The top electrode (1) was located closer to the ovaries, whereas the lowest electrode (6) was closer to the cervix. The events appeared approximately every 50 s and mainly propagated from the ovarian end to the cervical end of the uterine horn. (c) The high‐frequency components of the traces from (b). (d) The low‐frequency components of the traces from (b).

**FIGURE 3:**
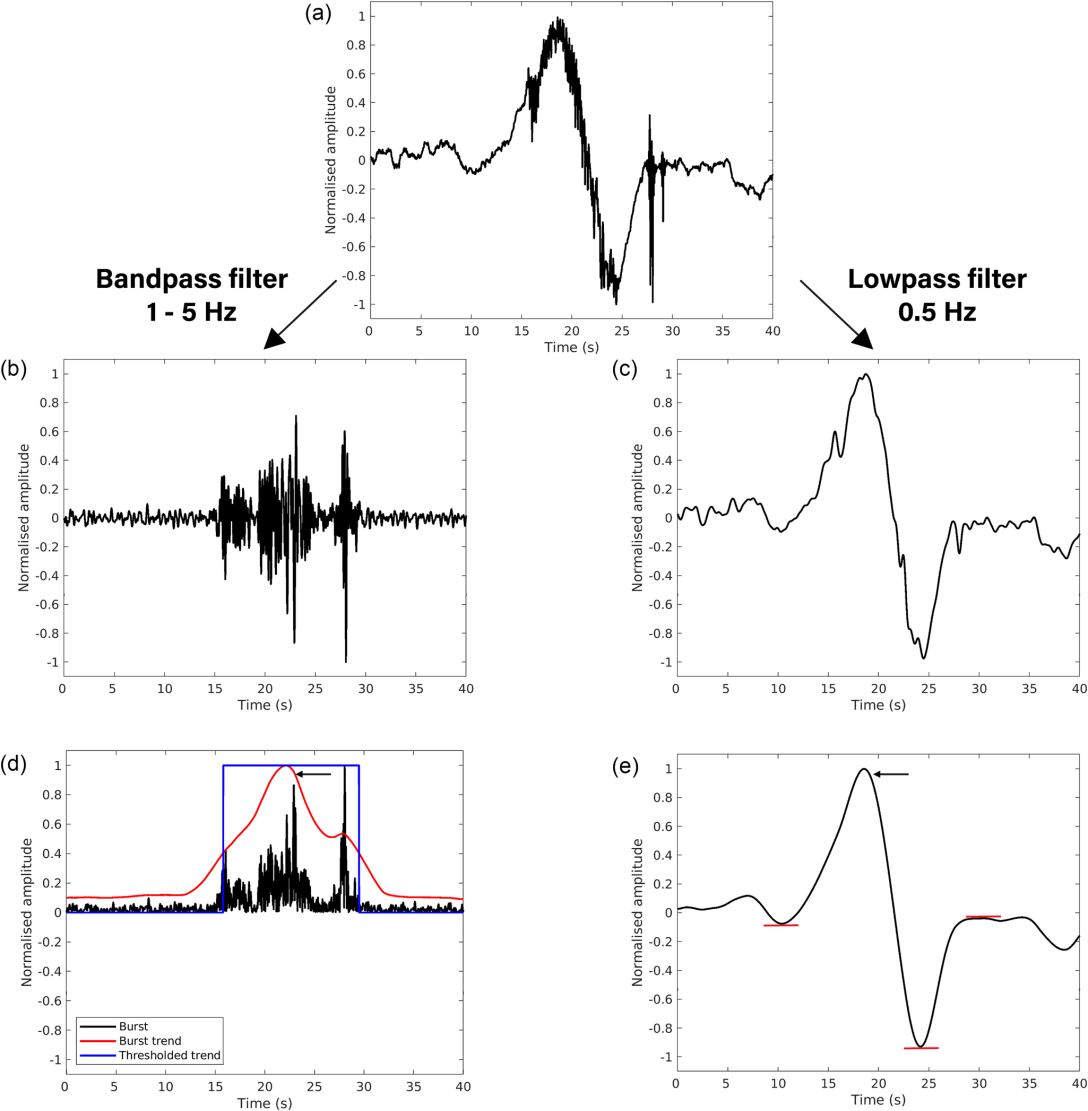
Example of the estimation of the duration of the slow wave and burst components. (a) The raw signal before filtering, centred around the electrical event. (b, c) The burst (b) and slow wave (c) components of the event. (d) The traces used to determine the duration of the burst. The segment of the trend greater than the threshold was defined as the duration of the burst if it included the peak associated with the burst. (e) The trend of the slow wave and the elements used to determine the duration of the slow wave. The red bars show the signal regions with a gradient equal to 0, which were used to determine the duration of the slow wave. The duration was determined to be the time between the initiation of the event and the return to baseline. The black arrows marked the peak associated with the event.

For each experiment, sections of 300 s of recording were analysed using the Gastrointestinal Electrical Mapping Suite (GEMS) (Yassi et al., 2012). The sections were selected from the pair of electrodes on either the ventral or the dorsal side based on a visual assessment of background noise. Two electrode arrays (64 channels in total) with the lowest background noise were chosen for further processing. A moving median filter with a 10 s window was used to estimate the baseline drift. This estimate was subsequently removed from the raw signal (Paskaranandavadivel et al., 2011). Figure 2 displays an example of the signals. Figure 2a is a schematic diagram of the position of the electrode arrays on the uterine horn used to record the traces displayed in the other panels. The signals contained both high‐ and low‐frequency components (Figure 2c,d). The activation times of the slow waves of electrical events were detected by the position of the maximum negative gradient of the signal in GEMS. Channels that exhibited a large amount of noise or artefact (e.g., due to motion) or that did not display any events, likely due to poor contact, were removed from analysis. Each event was subsequently analysed using MATLAB (version 2022b, MathWorks Inc., Natick, MA, USA). Activity was observed about 20 s before and after activation times; hence, a 40 s window of signal centred around the activation time was extracted for each analysed channel. From the windowed signals the duration of the slow wave (Figure 3c) and bursts (Figure 3b) was extracted to quantify the electrical activity at different stages of the oestrous cycle.

The two‐dimensional electrode arrays allowed the extraction of propagation velocity and direction of the events along the horn. Velocity values were calculated for each channel using a smoothed finite difference scheme described in Paskaranandavadivel et al. (2012). The propagation velocity was estimated by averaging the velocity values of each channel. The direction was determined by computing the mean of the *y* components from the velocity vectors in each column of the electrode arrays. The propagation direction was divided into three categories: (i) ovaries to cervix if all the mean values were positive, (ii) cervix to ovaries if all the mean values were negative, and (iii) other in cases with mixed signs or if 30% of the *y* components was of a different sign compared to the mean value.

The events were filtered with a bandpass filter with cutoff frequencies of 1 and 5 Hz to extract the high‐frequency (bursting activity) and a lowpass filter with a cutoff frequency of 0.5 Hz to extract the low‐frequency (slow wave) components of the signal, respectively. An example of the traces and the two components is presented in Figure 3. These filter limits removed respiration and heart rate frequencies while maintaining the signals of interest. The duration of the slow wave and the burst was estimated independently. Figure 3 shows an example of how the duration of both components was calculated from the windowed signal (Figure 3a). To measure the duration of the burst component in the signal (Figure 3b), the trend of the absolute value of the signal was computed using singular spectrum analysis and normalised (black and red traces in Figure 3d). The resulting trace was binarised using a threshold calculated with the Otsu (1979) method (blue trace in Figure 3d). The peak in the trend closest to the activation time was identified (black arrow in Figure 3d). If the segment where the signal was greater than the threshold included this peak, then the duration of the burst was determined to be the length of the segment. Otherwise, the signal was considered not to contain a burst; in these cases the high‐frequency signal did not have a significant or prolonged variation in magnitude from the mean signal. To measure the duration of the slow wave component (Figure 3c), the trend of the signal was computed using singular spectrum analysis and normalised (Figure 3e). First, the peak closest to the activation time was identified (black arrow in Figure 3e). The initiation of the event was identified as the moment with no gradient before the peak. The return to baseline was identified as the second moment with no gradient after the peak; the first moment indicated the event minimum before the return to baseline. The moments where the gradient was 0 are indicated with red bars in Figure 3e. The duration of the slow wave was defined as the time between the initiation of the event and the return to the baseline. Robustness of the duration estimation algorithms was tested by randomly selecting events and manually determining the duration of the event. A total of 25 events spread out over the oestrous cycle were analysed manually. The average difference between the manual and automated durations was 1.1 ± 3.9 s for the slow wave and 1.2 ± 2.0 s for the burst events.

### 2.5 Statistical analysis

Statistical analysis was performed in R using an analysis of variance. Statistical differences were calculated between the different stages of the oestrous cycle for all computed metrics except the propagation direction. The normality of the data was tested using a Shapiro–Wilk test. Significance was defined as a *P*‐value < 0.05. The full list of *P*‐values is presented in Table 1. Results are presented as means ± SD.

**TABLE 1:** List of *P*‐values.

	Pro‐oestrus–oestrus	Pro‐oestrus–metoestrus	Pro‐oestrus–dioestrus	Oestrus–metoestrus	Oestrus–dioestrus	Metoestrus–dioestrus
**Event interval (** **Figure** **5**)	0.805	0.004*	0.026*	0.001*	0.186	< 0.001*
**Propagation velocity (** **Figure** **6**b)	0.060	0.005*	0.999	0.697	0.064	0.005*
**Slow‐wave duration (** **Figure** **7**a)	0.922	0.999	0.893	0.957	1.00	0.937
**Burst duration (** **Figure** **7**b)	0.907	0.741	0.965	0.991	0.996	0.944
**Burst presence (** **Figure** **8**)	0.171	0.625	0.993	0.728	0.110	0.468

Bold and asterisk indicates significance.

## 3 RESULTS

Rhythmic electrical activity was successfully recorded from all 19 rats. The size of the arrays covered 85 ± 8% of the length of the uterine horn. An average of 56 ± 7 electrode channels out of 64 were used for processing the events in each experiment after discarding the channels with poor signal to noise ratios. The signals contained on average 1.26 ± 0.23 events per minute (0.021 ± 0.004 Hz).

**FIGURE 4:**
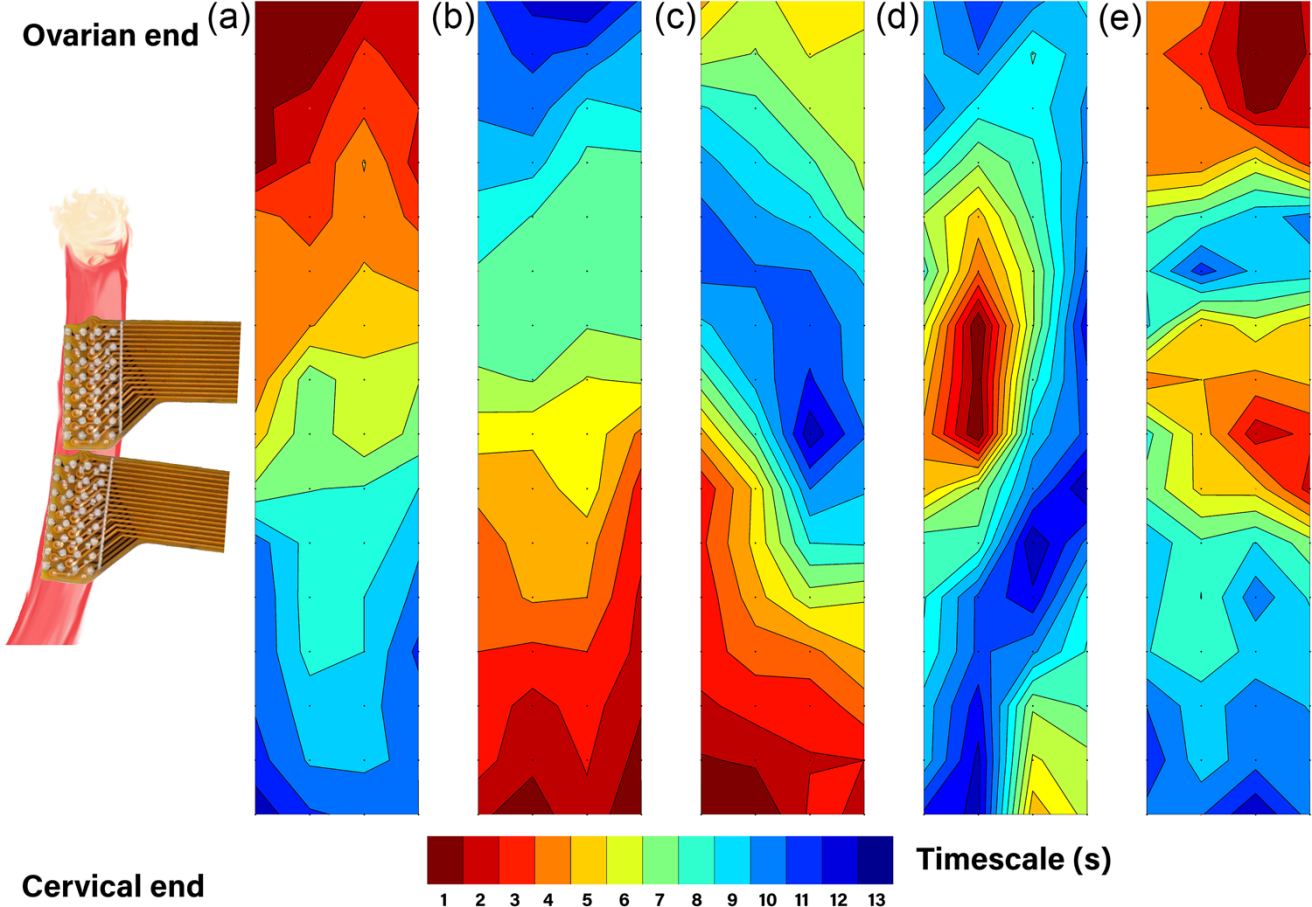
Examples of isochronal activation maps exhibiting events propagating in different directions from different rats over the course of the oestrous cycle. The regions are colour‐coded based on the activation times where early activation is coloured red and late activation is coloured blue. (a) The propagation of an event initiated near the ovaries and terminated at the cervix. (b) The opposite, an event initiated near the cervix and propagating to the ovaries. (c–e) Representative examples of activity that was classified as other. (c) An example of opposing propagation where two events were seen originating at the cervical and to ovarian ends and collide near the centre of the horn. (d) An event that originated in the centre of the horn and propagated in both directions. (e) Some more disorganised activity with various origin centres for the activity.

Figure 4 shows isochronal activation maps illustrating the different propagation types of electrical events in the uterine horn that were observed in different animals at different stages of the oestrous cycle. Regions are colour‐coded based on the activation times, where red represents early activation and blue represents late activation. The event displayed in Figure 4a originated near the ovaries and propagated down towards the cervix. In contrast, in Figure 4b, the event originated near the cervix and propagated towards the ovarian end of the horn. Figure 4c–e are examples of other types of activity. In Figure 4c, an opposing propagation pattern is shown where two events originated at either end and collided near the centre of the horn. Figure 4d shows an event that originated in the centre of the horn and propagated in both directions. Finally, Figure 4e displays some more disorganised activity with various origin centres for the activity.

**FIGURE 5:**
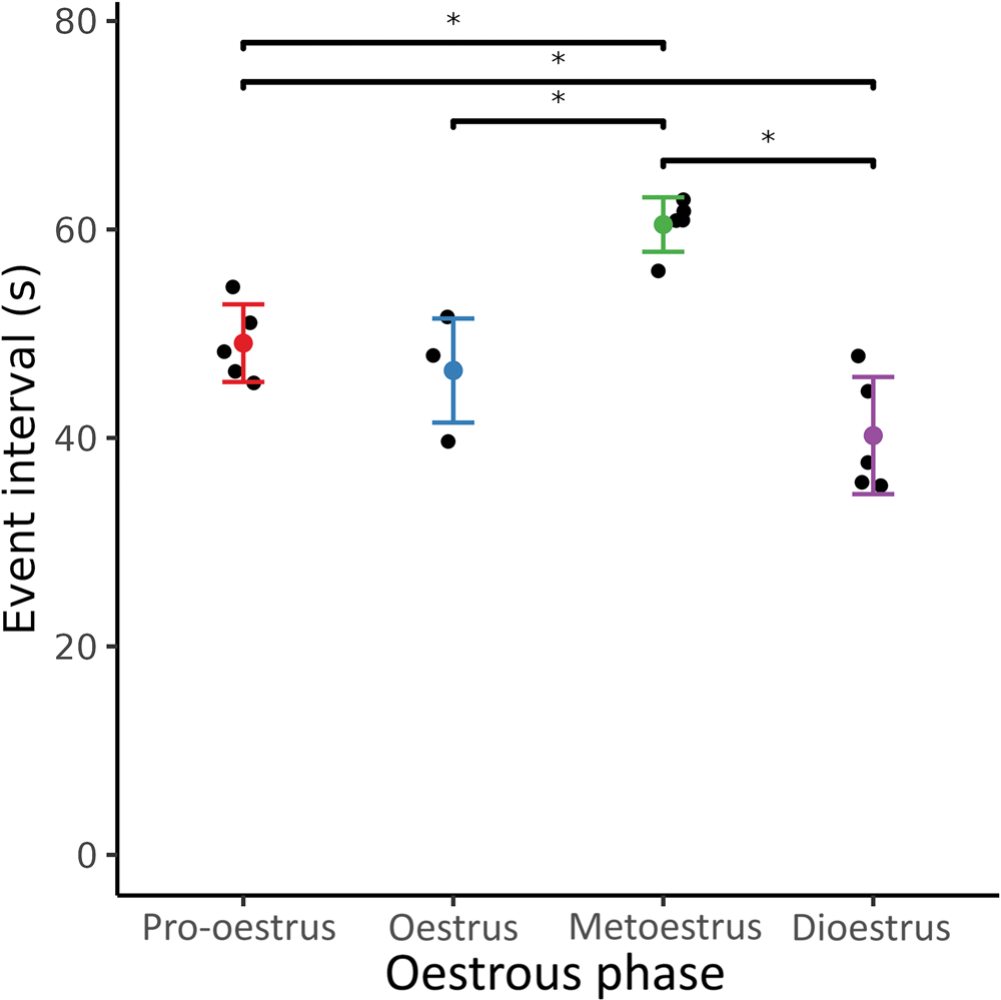
Interval between events at different stages of the oestrous cycle from 19 rats (*n* = 4 in oestrus, and *n* = 5 in the three other stages). The event interval changed significantly during the oestrous cycle. The dioestrus phase had the shortest interval, almost twice as small as during the metoestrus phase. The oestrus and pro‐oestrus phases had similar intervals between events. Error bars represent standard deviation, * *P* < 0.05.

Figure 5 shows the interval between events at different stages of the oestrous cycle. Each black point is the result of one experiment and the coloured points show the mean value of the at each stage of the oestrous cycle with an error bar representing the standard deviation. The event interval changed significantly during the oestrous cycle. It was smallest during the dioestrus phase at 40.2 ± 5.6 s (1.58 ± 0.25 cpm) and longest during the metoestrus phase at 60.5 ± 2.6 s (1.01 ± 0.05 cpm). The difference in the average interval between the oestrus and pro‐oestrus phases was not significant (*P* = 0.805), with intervals of 46.5 ± 5.0 s (1.32 ± 0.14 cpm) and 49.1 ± 3.7 s (1.29 ± 0.09 cpm), respectively. The interval difference between pro‐oestrus and both metoestrus and dioestrus was significant (*P* = 0.004 and 0.026, respectively), and the difference in intervals between metoestrus and both oestrus and dioestrus was significant (*P* = 0.001 and *P* < 0.001, respectively). The complete list of *P*‐values can be found in Table 1.

**FIGURE 6:**
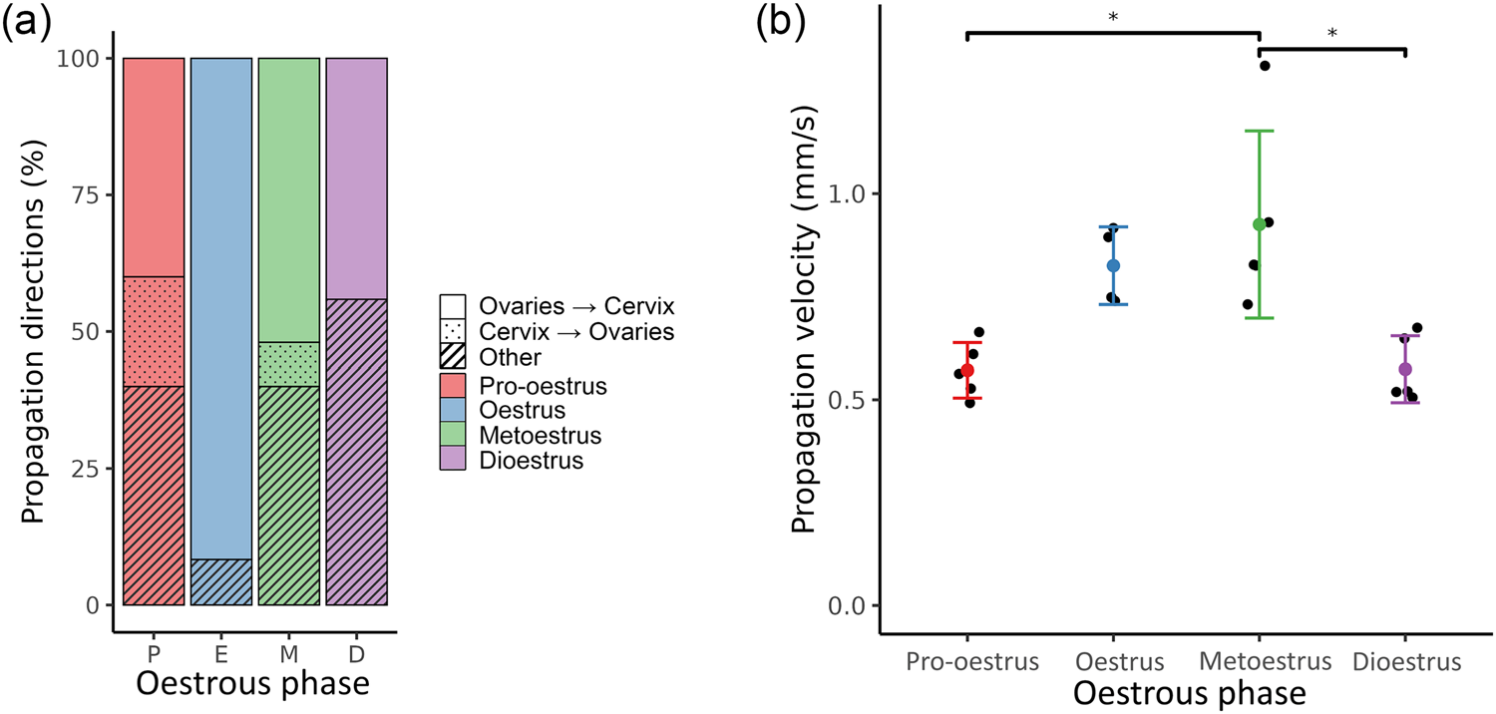
Propagation properties of the events at different stages of the oestrous cycle from 19 rats (*n* = 4 in oestrus, and *n* = 5 in the three other stages). (a) The direction of the events. Colours represent the oestrous phase, and shading represents the direction of propagation. (b) The propagation velocity at different stages of the oestrous cycle. The metoestrus phase had the highest propagation velocity and was significantly different from the velocities of the pro‐oestrus and dioestrus phases (*P* = 0.005 and 0.005, respectively). Error bars represent standard deviation; * *P* < 0.05.

Figure 6 presents the propagation properties at different stages of the oestrous cycle. Figure 6a displays the propagation direction of the events for each stage of the cycle. The dominant propagation direction changed over the course of the oestrous cycle. In pro‐oestrus, ovarian–cervical and other propagation types were both equally observed (40%). The oestrus and metoestrus phases exhibited events mainly propagating in the ovarian–cervical direction (92% and 52%, respectively). In the dioestrus phase, the main propagation direction was other (56%). Events propagating in the cervical–ovarian direction were only observed in pro‐oestrus and metoestrus (20% and 8%, respectively). Figure 6b shows the propagation velocity of the electrical events. The dioestrus and pro‐oestrus phase exhibited similar and slow propagation velocities respectively at 0.57 ± 0.08 and 0.57 ± 0.07 mm s^−1^ whereas both oestrus and metoestrus displayed more rapid propagation of events at 0.82 ± 0.09 and 0.93 ± 0.23 mm s^−1^, respectively. The metoestrus velocity was significantly different from the dioestrus (*P* = 0.005) and pro‐oestrus velocities (*P* = 0.005). The complete list of *P*‐values can be found in Table 1.

**FIGURE 7:**
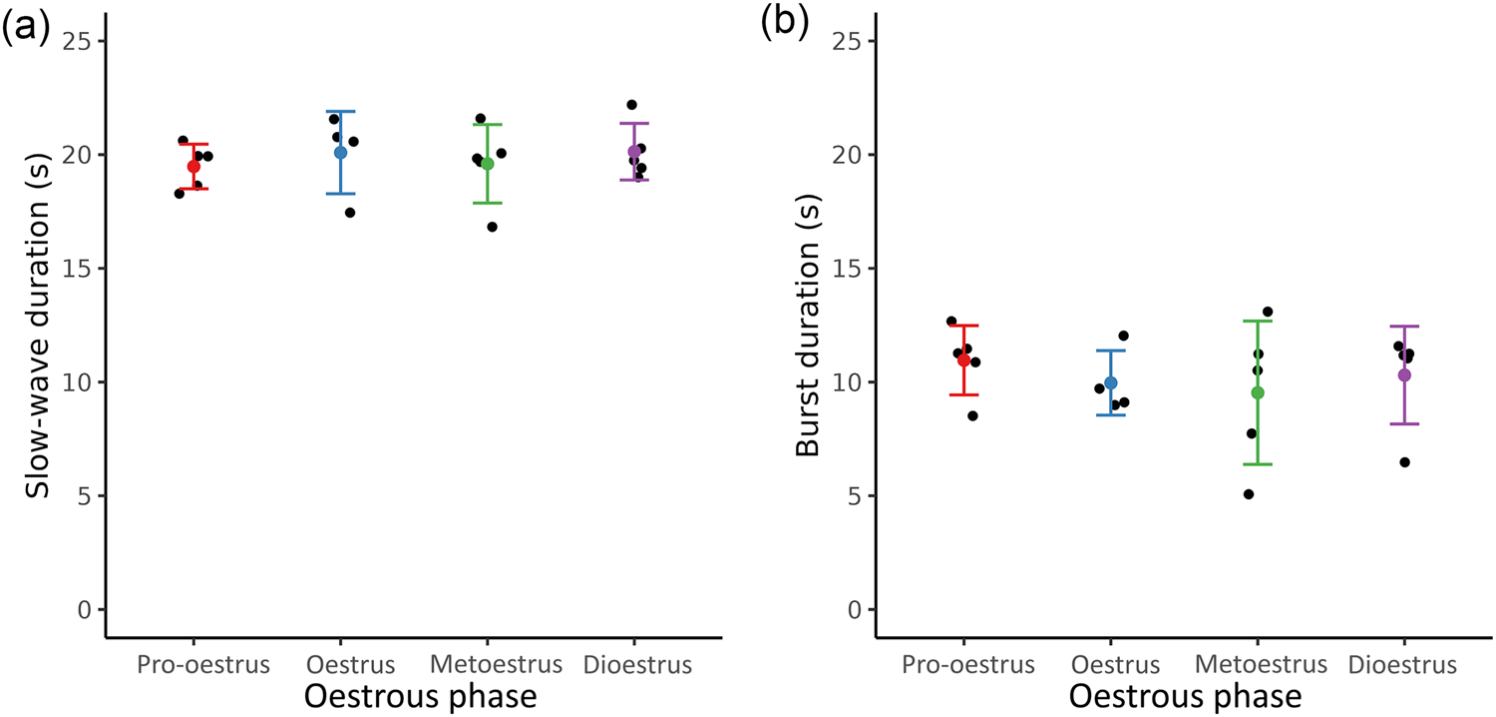
Slow‐wave (a) and burst (b) duration at different phases of the oestrous cycle for 19 rats (*n* = 4 in oestrus, and *n* = 5 in the three other stages). The duration of the slow wave was similar in all stages of the oestrous cycle lasting on average 19.9 ± 2.2 s. The burst duration was much shorter than the slow wave with an average of 10.2 ± 3.0 s. Error bars represent standard deviation.

Figure 7 displays the duration of the slow wave (Figure 7a) and bursts (Figure 7b) components of electrical events. In all stages of the oestrus, the duration of the slow waves was similar, with an average duration of 19.9 ± 2.2 s. The bursts were consistently shorter than the slow waves and had limited variations between stages with an average duration of 10.2 ± 3 s.

**FIGURE 8:**
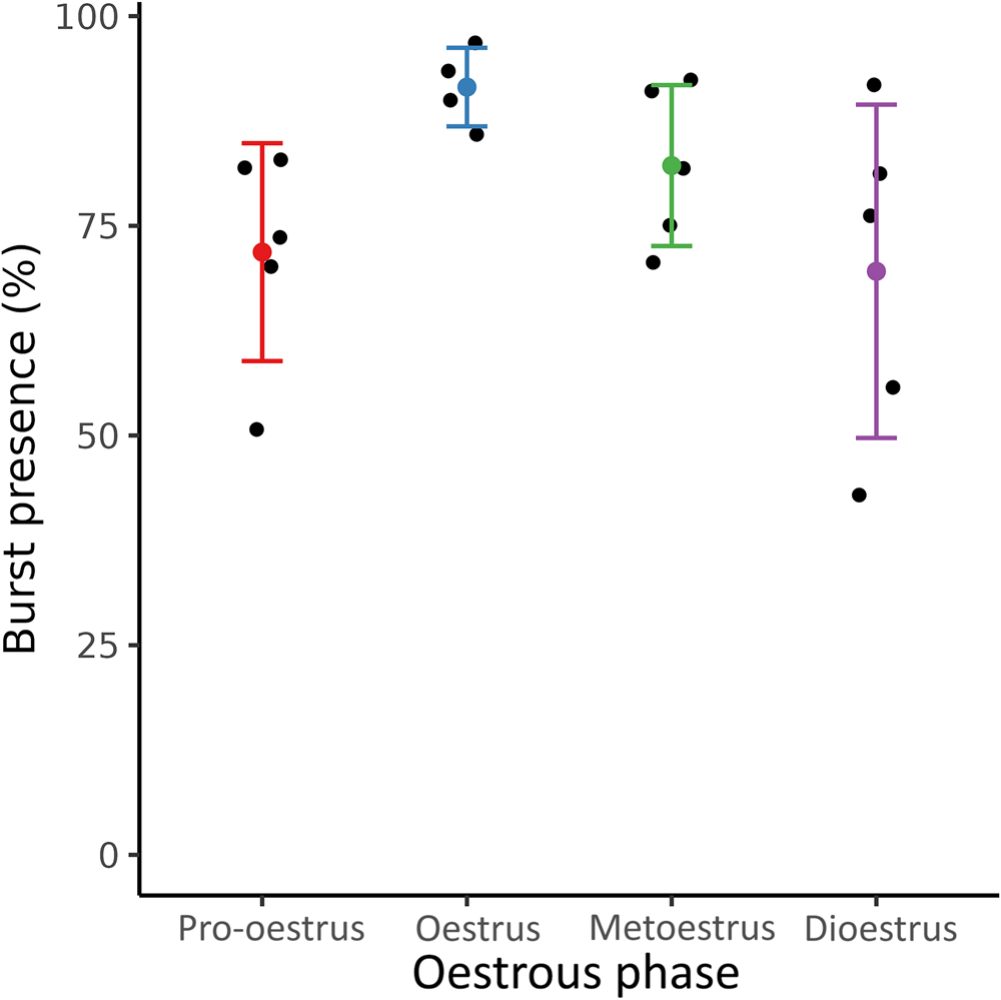
Percentage of slow waves exhibiting bursts at different stages of the oestrous cycle (*n* = 4 in oestrus, and *n* = 5 in pro‐oestrus, metoestrus, and dioestrus). The metoestrus and oestrus phases exhibited similar levels of bursting activity. In contrast, both pro‐oestrus and dioestrus phases exhibited a lower percentage of high‐frequency activity. Error bars represent standard deviation.

Not every event exhibited both bursts and slow wave activity simultaneously. Figure 8 displays the percentage of electrical events that included a high frequency burst of activity during the oestrous cycle. The pro‐oestrus and dioestrus phases exhibited the least high‐frequency activity with an average of 72 ± 13 and 70 ± 20% of events showing bursts, respectively. Most events in the oestrus and metoestrus phases were accompanied with bursting activity with an average of 92 ± 5% and 82 ± 10%, respectively.

## 4 DISCUSSION

Electrical signals of rat uteri were measured *in vivo* in all four stages of the oestrous cycle with high spatial resolution electrode arrays. The identified events were used to quantify the characteristics of uterine electrical activity across the oestrous cycle. Several propagation patterns were identified over the course of the oestrous cycle, but the most common propagation direction was from ovaries to cervix (Figure 6a). Similar patterns to those observed in this study (cervix to fundus, fundus to cervix, colliding waves and disorganised patterns) have previously been identified in human participants using transvaginal ultrasound (van Gestel et al., 2007). Electrical events were also tracked along the horn to measure the propagation velocity (Figure 6b). Significant differences in propagation velocity were identified when comparing the metoestrus phase with the pro‐oestrus and dioestrus phases, both with *P*‐values of 0.005. The signals were filtered using a bandpass and a lowpass filter to extract the bursting activity and the slow waves that have been previously observed (Garrett, Roesler et al., 2024) to quantify the duration of the events in both components. Not all events displayed bursting activity; hence, the percentage of events displaying bursts was calculated (Figure 8). The pro‐oestrus and dioestrus phases displayed fewer bursts than the oestrus and metoestrus phases. The high‐frequency bursts may be associated with mechanical activity (Wray & Prendergast, 2019), whereas slow waves can be present without exhibiting contractions. Hence, the pro‐oestrus and dioestrus phases may be more heavily associated with quiescence. This is consistent with the previous reports of the uterus being very active during the reproduction window (oestrus) and quieter during reproductive inactivity (dioestrus) (Freeman, 2006).

The electrical activity from the *in vivo* rat uterus has been studied previously (Crane & Martin, 1991; Ishikawa & Fuchs, 1978; Talo & Kärki, 1976). However, those studies only recorded with three to six implanted electrodes in conscious animals. Additionally, the oestrous cycle stages were classified differently, and thus direct comparisons between those studies and this one are difficult. Nevertheless, all three studies quantify similar metrics. The dioestrus phases consistently displayed a smaller number of events, except in Crane & Martin (1991) where the dioestrus phase exhibited the highest number of events. The results presented in this study also indicate that the dioestrus phase displayed the largest number of events by having the smallest inter‐event interval (Figure 5). In contrast, the metoestrus phase displayed significantly longer periods of quiescence compared to the other three stages, with *P* = 0.004, 0.001 and *P* < 0.001 for the pro‐oestrus, oestrus and dioestrus phases, respectively.

Most studies of uterine electrophysiology have focused on bursting activity (Domino et al., 2018, 2016; Lammers et al., 2015; Lammers & Hamid, 1998; Lutton et al., 2018; Rutkevich et al., 2025). These burst signals in other organs have been shown to represent mechanical contractions (Kuruppu et al., 2022) and this is the common interpretation in uterine tissue. They may be related to intracellular ion transport related to contractions. The average duration of the bursts (Figure 7b) of 10.2 ± 3.0 s reported here was consistent with previous studies which measured burst durations of 10–12 s at different stages of the oestrous cycle (Crane & Martin, 1991; Ishikawa & Fuchs, 1978; Talo & Kärki, 1976). They also report bursts lasting between 1 and 5 s, which were not observed in the present study. Rutkevich et al. (2025) reported burst durations significantly longer, ranging between 27 and 40 s. Nevertheless, the duration of the events measured here corresponded with most of the previously reported measures. The discrepancies between previous studies and the findings presented here might result from the use of anaesthesia during the recording periods, which has been found to alter the electrical activity in the stomach (Aghababaie et al., 2023). Previous studies sutured the electrodes in the uterus and rats were allowed to move freely during the recording periods. Since other methods of recording disrupt regular activity (Anderson et al., 2024; Crane & Martin, 1991), it is likely that long‐term recordings with sutured electrodes may have also affected uterine activity in these studies.

Slow waves reflect shifts of membrane potential at the tissue scale, over a time period of multiple seconds. These slow waves in other tissues are not always associated with burst activity (Kuruppu et al., 2022), leading to the hypothesis that slow waves that meet a certain threshold lead to contraction. In a pilot study of non‐pregnant uteri, there has previously been a one‐to‐one correspondence between slow waves and burst activity (Garrett, Roesler et al., 2024); however, in this study we observed slow waves more often than bursting activity (with the lowest rates of bursting in pro‐oestrus and dioestrus phases) aligning with previous observations of limited to no bursts in late dioestrus to early pro‐oestrus (Talo & Kärki, 1976). The duration of slow waves (Figure 7a) has not been previously measured, but it was consistently longer than the fast waves, typically lasting 19.9 ± 2.2 s (approximately 2 times longer). The slow wave may be a tissue level mechanism that helps synchronise uterine activity by initially depolarising a tissue region, but the bursts are more likely to reflect cellular activity related to a mechanical contraction. The slow wave triggers an initial depolarisation, and if the region is large enough bursts are observed. The bursts subside as the slow wave hyperpolarises and the tissue returns to a resting potential. This sequence of events, not dissimilar to the one which occurs at a cellular level (Wray & Prendergast, 2019), helps explain the longer duration of slow waves and the absence of bursts in some cases. This type of bursting behaviour has been observed in gastrointestinal smooth muscle (Kuruppu et al., 2022; Lammers & Slack, 2001); however, gastrointestinal slow waves originate from an interstitial cells of Cajal pacemaker network, whereas the origin of electrical activity in the uterus has yet to be determined (Wray & Taggart, 2026). Bursts may be related to calcium transport; however, calcium measurement was outside the scope of this study. Simultaneous measurements in the future may provide the valuable link between electrophysiological recordings and mechanical activity (Burdyga et al., 2009).

Most records of propagation velocity come from studies done during pregnancy (Rabotti & Mischi, 2015) and most studies on non‐pregnant uterine electrical activity do not measure it (Crane & Martin, 1991; Ishikawa & Fuchs, 1978; Rutkevich et al., 2025; Talo & Kärki, 1976). Nevertheless, Garrett, Roesler et al. (2024) reported the propagation velocity in one rat to be 1.59 ± 0.37 mm s^−1^ and an early study from Bozler (1938) reported propagation velocities in non‐pregnant guinea pigs in the range 1–3 mm s^−1^. Both of these values are similar to the ones measured in this study, which ranged between 0.49 and 1.13 mm s^−1^ over the course of the oestrous cycle. It is interesting to note that in most of the studies conducted during pregnancy, the propagation velocity is several orders of magnitude greater than measured in this study with, for example, a range of 5–130 mm s^−1^ in humans during labour (Rabotti & Mischi, 2015). This difference might be related to the different size of the uterus between species, as the human uterus is significantly larger than the rodent uterus (Garrett et al., 2022). There are also size differences between pregnant and non‐pregnant uteri, and there is evidence that an increased number of gap junctions during labour would have the effect of improving the coupling between cells and the propagation of electrical activity (Malik et al., 2021; Miller et al., 1989).

Different propagation patterns were observed during the cycle (Figure 4) and used to quantify the propagation direction of events (Figure 6a). The primary propagation direction was from ovaries to cervix in the oestrus and metoestrus phases, which is similar to reports from previous studies (Crane & Martin, 1991; Talo & Kärki, 1976). Some retrograde propagation, that is, from cervix to ovaries, was observed in both pro‐oestrus (20%) and metoestrus (8%) but was absent in dioestrus and oestrus (Figure 6a). Other patterns were observed where two events initiated simultaneously at both ends of the horn and collided in the centre of the horn or an initiation region appeared in the centre of the horn and the electrical wave propagated in all directions. Ishikawa & Fuchs (1978) identified events that started in the centre of the horn and events that propagated in a disordered manner. Similar patterns were also observed in this study and were classified as *other* types of propagation patterns, which were the dominant type of propagation in the dioestrus phase. The dioestrus phase is a resting phase, which might explain why the activity is not as organised as in the oestrus phase (mating). In contrast, the pro‐oestrus and metoestrus phases are more transient and the two shortest phases of the cycle (12 and 6–8 h, respectively) and the propagation patterns are transitioning towards the organisation of the oestrus phase or the disorganisation of the dioestrus phase. These types of propagation patterns have been observed previously in humans and classified by van Gestel et al. (2003). The contractions that are generated by electrical activity are thought to contribute to uterine function (Kunz & Leyendecker, 2002). One of their roles may be to aid sperm reach the oviducts to fertilise the oocytes supported by the dominant cervico‐fundal direction of contractions in humans during the peri‐ovulatory phase (Wang et al., 2024). However, during the oestrus phase of the cycle, when rats mate, the electrical events predominantly propagate from the ovaries to the cervix, which opposes the motion of sperm. This behaviour has been observed in conscious rats (Crane & Martin, 1991; Ishikawa & Fuchs, 1978; Talo & Kärki, 1976) and in this study. This is different from what has been suggested in humans (Wang et al., 2024), which may relate to differences in litter size and uterine anatomy/function between humans and rodents (Garrett et al., 2022). The human uterus is approximately five times longer than the rat uterus relative to body size (Roesler et al., 2025; Tsilchorozidou & Conway, 2004) and rodent sperm twice as large as human sperm (Cummins & Woodall, 1985). Thus, in humans, contractions might be necessary to help sperm motility to increase the likelihood of fertilisation, but not in rodents.

The mechanism that drives the occurrence of coordinated uterine electrical activity is still an open question and the search for a pacemaking cell or region that generates electrical waves like in the gastrointestinal tract is still ongoing (Wray & Taggart, 2026). The results in this paper indicated that the origin of the electrical activity was never located in a single fixed region during the oestrous cycle. Initiation sites were primarily located near the ovaries, but also appeared regularly at the cervix and sometimes in the centre of the horn (Figure 6a). This is further evidence that a single pacemaking region is not present in the uterus and another mechanism is driving the initiation of electrical events (Wray & Taggart, 2026). Telocytes, also referred to as interstitial Cajal‐like cells, have been identified as a possible key in the generation of electrical events in the uterus (Duquette et al., 2005). These passive cells are connected to several smooth muscle cells and might help maintain their resting membrane potential. Models suggest that electrically passive cells connected to active smooth muscle cells can generate action potentials without stimulation and might explain the appearance of initiation sites across the uterine horns (Means et al., 2025; Xu et al., 2015). Gap junctions might locally increase the connectivity between telocytes and smooth muscle cells enough to drive uterine regions into generating electrical activity. The number of gap junctions increases near the onset of labour and plays a crucial role in successful parturition (Puri & Garfield, 1982). They are sensitive to hormonal influences, and the changes in concentration of sex hormones during the oestrous cycle might allow different regions to be active at different times. Imaging techniques using confocal microscopy have been used to quantify the density of gap junctions in the uterine horns at different times of the oestrous cycle to investigate their role in the generation of electrical events (Garrett, Prince et al., 2024).

Considering the electrical‐based metrics alone is insufficient to determine the stage of the oestrous cycle, but when viewed together they bring to light trends in the electrical activity that characterize the different phases. The oestrous cycle is a continuous phenomenon that can be divided into four distinct categories. Although this categorisation is practical, it does not account for periods when a rat might be transitioning from one phase to the next. In those cases, the electrical properties might lie between the expected values for each stage. Hence, some of the metrics used to quantify rat uterine electrical activity, notably the burst presence and duration, display trends and differences that are not statistically significant. For example, the number of bursting events observed in the pro‐oestrus and dioestrus phases appears to be lower than the ones from the oestrus or metoestrus phase. If a rat is transitioning from metoestrus to dioestrus, the events might contain more bursts but propagate at dioestrus‐level velocities. The inclusion of transition phases will help refine the trends that are seen in the data and observe with more detail the ebbs and flows of uterine activity during the oestrous cycle. Further investigation of these trends would make it possible to determine the stage of the oestrous cycle based on the characteristics of the electrical activity in the uterus.

### 4.1 Perspectives and significance

The results presented in this study confirmed that the electrical activity changes over the course of the oestrous cycle. Results were in accordance with those from previous reports and provided additional metrics to characterise uterine electrical activity. Notably, the spatial patterns observed indicated that spontaneous activity preferentially originated near the ovaries, but the dominant origin of the activity changed over the course of the oestrous cycle. Electrical events always contained a low‐frequency component; however, not all of them exhibited high‐frequency bursting activity. In the scope of human applications, this suggests that it is important to consider electrical activity alongside the mechanical contractions, as departures from expected behaviour in the electrical propagation and coordination could be indicative of uterine dysfunction.

## AUTHOR CONTRIBUTIONS

All authors conceived and designed the research. Mathias W. Roesler, Leo K. Cheng, and Amy S. Garrett performed the experiments. Mathias W. Roesler, Leo K. Cheng, Amy S. Garrett, and Alys R. Clark analysed the data and interpreted the results. The manuscript was drafted by Mathias W. Roesler. All authors edited and revised the manuscript. All authors have read and approved the final version of this manuscript and agree to be accountable for all aspects of the work in ensuring that questions related to the accuracy or integrity of any part of the work are appropriately investigated and resolved. All persons designated as authors qualify for authorship, and all those who qualify for authorship are listed.

## CONFLICT OF INTEREST

L.K.C. has intellectual property in the field of gastrointestinal electrophysiological mapping and is a shareholder in FlexiMap Ltd. The other authors declare that the research was conducted in the absence of any commercial or financial relationships that could be construed as a potential conflict of interest.

## Data Availability

All the code used for analysis is available on GitHub at https://github.com/virtual‐uterus/uterine‐electrophysiology. Experimental data can be made available upon request.
